# Community engagement in health systems interventions and research in conflict-affected countries: a scoping review of approaches

**DOI:** 10.1080/16549716.2022.2074131

**Published:** 2022-06-28

**Authors:** Anna Durrance-Bagale, Manar Marzouk, Lam Sze Tung, Sunanda Agarwal, Zeenathnisa Mougammadou Aribou, Nafeesah Bte Mohamed Ibrahim, Hala Mkhallalati, Sanjida Newaz, Maryam Omar, Mengieng Ung, Ayshath Zaseela, Michiko Nagashima-​Hayashi, Natasha Howard

**Affiliations:** a Saw Swee Hock School of Public Health, National University of Singapore and National University Health System, Singapore; b Department of Global Health and Development, London School of Hygiene and Tropical Medicine, London, UK; c Stanford Distinguished Careers Institute, Campus Drive, Stanford, CA, USA; d Department of Community Health Sciences, Rady Faculty of Health Sciences, University of Manitoba, Winnipeg, Manitoba, Canada; e Chelsea and Westminster Hospital NHS Foundation Trust, London, UK

**Keywords:** Health systems, community engagement, co-creation, people-centred healthcare, conflict

## Abstract

**Background:**

Healthcare research, planning, and delivery with minimal community engagement can result in financial wastage, failure to meet objectives, and frustration in the communities that programmes are designed to help. Engaging communities – individual service-users and user groups – in the planning, delivery, and assessment of healthcare initiatives from inception promotes transparency, accountability, and ‘ownership’. Health systems affected by conflict must try to ensure that interventions engage communities and do not exacerbate existing problems. Engaging communities in interventions and research on conflict-affected health systems is essential to begin addressing effects on service delivery and access.

**Objective:**

This review aimed to identify and interrogate the literature on community engagement in health system interventions and research in conflict-affected settings.

**Methods:**

We conducted a scoping review using Arksey & O’Malley’s framework, synthesising the data descriptively.

**Results:**

We included 19 of 2,355 potential sources identified. Each discussed at least one aspect of community engagement, predominantly participatory methods, in 12 conflict-affected countries. Major lessons included the importance of engaging community and religious leaders, as well as people of lower socioeconomic status, in both designing and delivering culturally acceptable healthcare; mobilising community members and involving them in programme delivery to increase acceptability; mediating between governments, armed groups and other organisations to increase the ability of healthcare providers to remain in post; giving community members spaces for feedback on healthcare provision, to provide communities with evidence that programmes and initiatives are working.

**Conclusion:**

Community engagement in identifying and setting priorities, decision-making, implementing, and evaluating potential solutions helps people share their views and encourages a sense of ownership and increases the likely success of healthcare interventions. However, engaging communities can be particularly difficult in conflict-affected settings, where priorities may not be easy to identify, and many other factors, such as safety, power relations, and entrenched inequalities, must be considered.

## Background

Health system planning, delivery, and research are generally predicated on what has gone before, with often minimal community involvement in any of these aspects. This can result in financial wastage, failure to meet objectives, and frustration within the communities that health systems were designed to support. Engaging communities – as individual service-users and user groups – in the planning and delivery of healthcare initiatives from inception allows people at least some control over their healthcare, and promotes transparency and accountability. Their engagement increases the likelihood that health policies and initiatives will be successful, and is a first step toward ensuring health system interventions adhere to human rights principles [1].

People-centred approaches, incorporating deliberative spaces for community involvement that support feedback mechanisms and equal representation by all groups within a community, at all stages of healthcare delivery, help ensure services will be context-specific. Community priorities may be different from those of experts, especially experts not familiar with community realities. Standard interventions designed and implemented through a top-down approach, from experts based in high-income countries with little understanding of the cultural context, can exacerbate pre-existing inequalities or be outright harmful, as seen during early phases of the Covid-19 pandemic, in which measures such as lockdowns and isolation increased incidence of harms such as gender-based violence and violence against children [2,3]. People-centred approaches can encourage use of services and successful programme implementation [1]. However, engagement in healthcare provision is unlikely to be the priority for many communities and individuals during conflict, so necessary trade-offs must be considered. Priorities must be selected, preferably by communities [4]. Coupled with this is the need to account for power relations, inequalities, and community cultural and social norms [5]. Co-design and delivery of effective health services is complex, even more so in conflict-affected settings where people may be uncomfortable coming forward to share their views.

Strengthening and assessing the performance of conflict-affected, fragile, and often fragmented health systems is essential to ensure effective service delivery and value for often constrained resources. In conflict-affected countries, health systems should ensure that interventions promote cohesion between communities and do not exacerbate existing issues, e.g. resource competition between host and displaced communities [6]. There must be a balance between protecting and addressing the needs of marginalised populations and ensuring others are not left behind. Literature on health systems in conflict-affected countries is expanding, and awareness of the importance of community engagement within health systems is also increasing. However, there are still many gaps in our knowledge of effective community engagement in health system interventions, particularly during acute and protracted conflict. Such knowledge is vital to ensuring interventions are relevant to community needs and priorities [1].

### Objectives

We thus aimed to identify and interrogate the literature on community engagement within health system interventions and research in conflict-affected settings. Objectives were to: (i) summarise the extent, nature, and distribution of existing health systems literature related to conflict-affected countries that involved any community engagement; (ii) synthesise main findings; and (iii) identify lessons for community engagement in future health systems interventions and research in conflict-affected countries.

## Methods

### Study design

We conducted a scoping review using Arksey and O’Malley’s six-stage scoping framework with Levac *et al*’s 2010 revisions and Khalil *et al*’s 2016 refinements [7–11]. A scoping review is a type of research synthesis aimed at examining the existing literature on a particular topic and allowing gaps, key concepts, and different sources of evidence to be identified [12]. Scoping reviews are useful for broad research questions or complex and heterogeneous literature not amenable to a more focused systematic review [9].

### Stage 1. Research question

Our research question was: ‘What is the scope (i.e. extent, distribution, nature) and main findings of the literature on community engagement in health system interventions or research in conflict-affected countries?’ Table 1 provides study definitions.

**Table 1.** Study definitions.

Community engagement	A dynamic relational process that facilitates communication, interaction, involvement, and exchange between an organization and a community for a range of social and organisational outcomes [13]
Co-creation	The process by which people collaboratively both define and implement a solution (i.e. co-design plus co-production) [14]
Co-design	Involves an attempt to define a problem and then define a solution, in an equal partnership of individuals who work within the system (e.g. healthcare staff), individuals with lived experience of using the system (e.g. service-users/carers) and ‘designers’ [14]
Co-production	Involves an attempt to implement a proposed solution in an equal partnership of individuals working within the system (e.g. healthcare staff), individuals with lived experience using the system (e.g. service-users/carers) and ‘designers’ (e.g. researchers/consultants) [14]
Conflict-affected country	Has produced more than 1,000 battle-related deaths over a ten-year period, or more than 200 battle-related deaths in any three-year period, as measured by the Uppsala Conflict Data Program [15]
Health system	Consists of all organisations, people, and actions whose primary intent is to promote, restore or maintain health, including efforts to influence determinants of health as well as more direct health-improving activities [16]
Participatory research	A broad term referring to research conducted ‘with and by’ participants rather than on them [17], participation can in reality include anything from involvement to collaborative action

### Stage 2. Identifying documents

To ensure breadth and comprehensiveness, we systematically searched five electronic databases: EMBASE, Global Health, Medline, Scopus, and Web of Science. We used the terms and related terminology for ‘conflict-affected situation’ (e.g. political conflict, war, political instability) AND ‘health system’ (e.g. healthcare system, health care system) AND ‘community engagement’ (e.g. community participation, involvement, action) adapted to subject headings for each database and Google Scholar. Table 2 provides an example of our search syntax.

**Table 2.** Search syntax and keywords for Medline.

Keywords	Medline
Conflict-affected situations	(1) ‘warfare and armed conflicts’/ or exp armed conflicts/ (2) Conflict* adj3 (affected or zone or zones or Armed or Political or Violent)).mp. (3) (War or Wars or Warfare or Rebel* or revolution* or Uprising* or Insurgen* or Complex emergenc*).mp. (4) (Political instability or Political unrest).mp. (5) 1 or 2 or 3 or 4
Health system	(6) ‘Delivery of Health Care’/ (7) Public Health Systems Research/ (8) (system* adj3 (care* or health* or healthcare*)).mp. (9) 6 or 7 or 8
Community engagement	(10) Community Participation/ or Community-Based Participatory Research/ (11) (Community* adj3 (action* or involve* or participat* or engag* or driven* or based)).mp. (12) (Public* adj3 (action* or involve* or participat* or engag* or driven* or based)).mp. (13) 10 or 11 or 12 (14) 5 and 9 and 13 (15) 5 and 9 and 14

### Stage 3. Selecting documents

We established eligibility criteria via an iterative process, and all authors agreed initial criteria based on research definitions and questions (Table 3). As shown in Tables 1 and 3, context was restricted to conflict-affected situations, topics were restricted to health system or components, outcomes were restricted to descriptions of community engagement approaches and implementation, source types were restricted to academic and technical literature, and language was restricted to documents with an English abstract and text in any language that researchers could extract and translate with the help of Google Translate. All study designs, interventions, and participants (e.g. health-workers, expert panels, service-users) were included.

**Table 3.** Eligibility criteria.

Criteria	Inclusion	Exclusion
1. Context	Includes a conflict-affected setting (e.g. peri-conflict, ‘post-conflict’).	Context is not a conflict-affected country, territory, or subnational setting.
2. Topic	Involves a national or subnational health system or health system component (e.g. governance, financing, workforce, medical products, information, service delivery).	Does not relate to a national or subnational health system or its components or anything about community engagement, participation, or co-design.
3. Outcomes	Describes community engagement, co-design, participatory approach or method/s or results.	Does not describe any community engagement approach, methods or results.
4. Source type	Includes primary research findings (e.g. qualitative, quantitative, mixed, case study).	Does not include primary research (e.g. opinion commentary, history, literature review, secondary analysis only).
5. Time-period	Published 2000+ and data were collected in 2000 or later.	Publication date or data collected before 2000.
6. Language	Any if an English abstract is available.	No English abstract.

**Figure 1.**
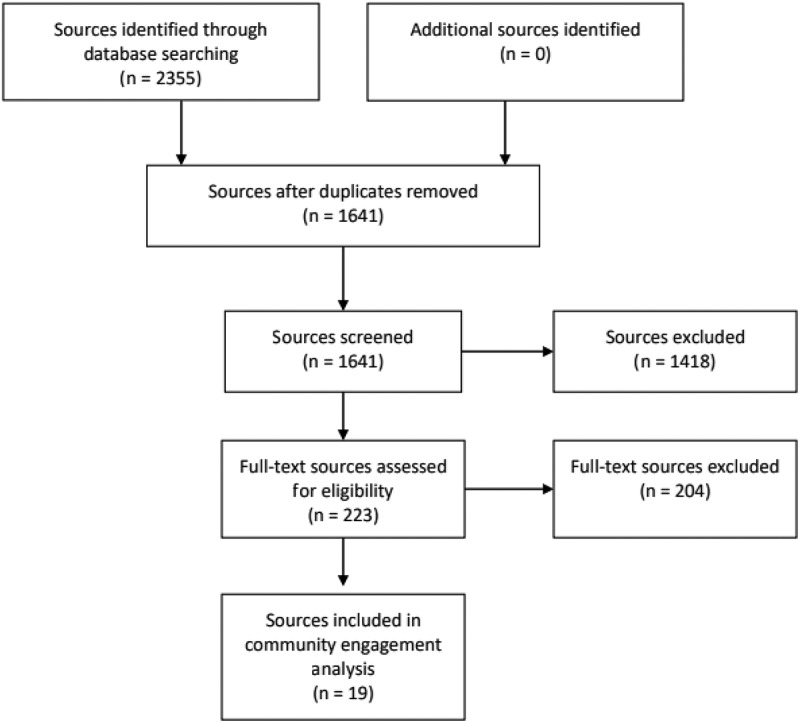
PRISMA flow diagram for scoping reviews.

Figure 1 provides the PRISMA flow diagram. First, we identified documents in databases and websites. Second, we removed duplicates using the reference manager Mendeley. Third, we screened titles and abstracts against eligibility criteria to remove obviously irrelevant documents using Rayyan software. Fourth, we screened full texts against eligibility criteria to remove additional ineligible documents.

### Stage 4. Extracting (charting) data

All authors contributed to extracting data to an Excel sheet using the following headings: (i) identifiers, i.e. lead author, publication year, source type (e.g. article, conference abstract, book, report); (ii) characteristics, i.e. country, study design (e.g. qualitative, quantitative, mixed-methods), participant characteristics; (iii) findings, i.e. methods used in community engagement, outcomes, lessons described.

### Stage 5. Collating, analysing, and reporting findings

We summarised the number of sources by extent (i.e. number, publication year, type), distribution (i.e. publication language, countries included), and nature (i.e. outcomes included, study design, participant or other characteristics). We then synthesised data descriptively, categorising sources as describing participatory methods, co-creation approaches, or both. We reported the main findings and discussed implications for policy, practice, and future research.

### Stage 6. Consulting stakeholders

We consulted four health systems researchers for their suggestions on draft findings and any additional sources we may have missed. This reassured us on our interpretations though it did not provide any additional eligible sources.

## Results

### Scope of the literature

#### Extent

**Figure 2.**
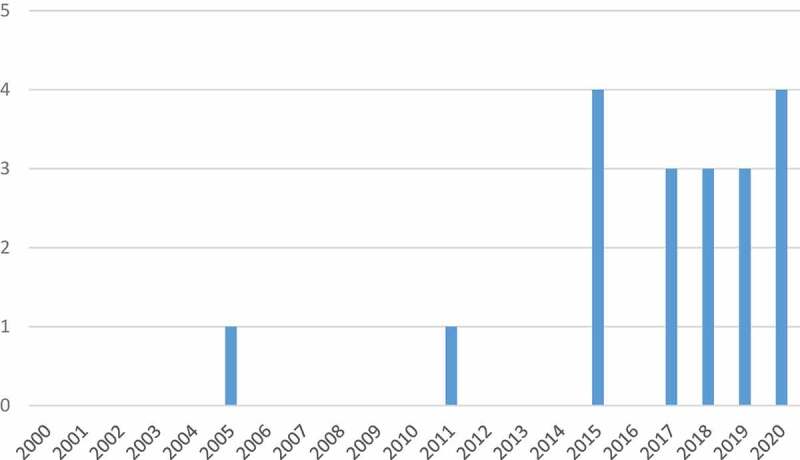
Documents by publications year.

We included 19 eligible documents of 2355 identified as potentially eligible (Figure 1; Table 4). Initially, we removed 714 duplicates. Of the remaining 1641, 1418 were excluded through title and abstract screening, and 204 through full-text screening. All eligible documents were in English. One (5%) was an abstract and 18 (95%) were peer-reviewed journal articles. We found a potential upward temporal trend, with none published 2000–2004, 2006, 2007–2010, 2012–2014, or 2016; four each (21%) in 2015 and 2020; three (16%) in 2017, 2018, and 2019; and one each (5%) in 2005 and 2011 (Figure 2).

**Table 4.** Sources and topics covered, ordered by lead author.

Lead author (year)	Reference	Country/ies	Participation	Co-design	Other
Abdullahi (2020) [18]	13	Nigeria	X		
Adams (2020) [19]	15	Sierra Leone	X		
Ager (2015) [20]	17	Nigeria	X	X	
Akseer (2019) [21]	23	Afghanistan			X
Anwari (2015)[22]	14	Afghanistan	X		
Baingana (2011)[23]	26	Uganda			X
Elmusharaf (2017) [24]	18	South Sudan	X		
Erismann (2019) [25]	16	South Sudan, Haiti	X	X	
Foster (2017) [26]	20	Myanmar, Thailand	X		
Ho (2015) [27]	21	DRC	X		
Kozuki (2018) [28]	27	South Sudan			X
Mandal (2005) [29]	19	South Sudan	X		
Rosenberg (2017) [30]	31	Liberia			X
Sami (2018) [31]	30	South Sudan			X
Saymah (2015) [32]	28	Palestine			X
Sengupta (2020) [33]	22	India		X	
Steven (2019) [34]	25	DRC			X
Tangseefa (2018) [35]	24	Myanmar			X
Valadez (2020) [36]	29	South Sudan			X

#### Distribution

Of 17 single-country and two multi-country documents, five (25%) focused on South Sudan; two (10%) each on Afghanistan, Democratic Republic of the Congo (DRC), and Nigeria; one (5%) each on India, Liberia, Myanmar, Palestine, Sierra Leone, and Uganda; and two (10%) included Haiti/South Sudan and Myanmar/Thailand.

#### Nature

Fourteen (74%) documents focused on community engagement in health system assessment and five (26%) focused on community engagement with relevance to at least one health system component (e.g. service delivery, governance). Ten (53%) documents used qualitative approaches, while five (26%) were mixed-method and four (21%) were entirely quantitative. Qualitative methods were primarily semi-structured interviews (14/15), followed by focus group discussions (4/15) and observations (3/15), while quantitative methods consisted primarily of cross-sectional surveys (6/9) and secondary analyses of routine data (3/9). Only seven (50%) of the 14 documents including engagement within health system assessment described using an analytical framework. Frameworks were heterogeneous, including the Balanced Scorecard and Community Scorecard, Consolidated Framework for Implementation Research (CFIR), TB REACH, WHO Assessment Instrument for Mental Health Systems (WHO-AIMS), and the people-centred health systems governance conceptual model (i.e. cultivating accountability, engaging with stakeholders, setting a shared strategic direction, stewarding resources responsibly).

### Community engagement approaches

Approaches to community engagement were relatively similar across documents, with nine (47%) including participatory methods generally, three (16%) explicitly discussing co-creation approaches, while nine (47%) included other engagement methods.

#### Participatory methods

Nine documents included participatory methods generally [18–20, 22, 24–27, 29]. Abdullahi *et al*’s description of tuberculosis and HIV outreach services for internally displaced populations in Northeast Nigeria, demonstrated that community-driven multi-stakeholder programs were able to increase healthcare access for displaced people living with tuberculosis and HIV and contribute to health system strengthening [18].

Anwari *et al*’s case study of a people-centred health systems governance intervention in Afghanistan showed a participatory approach encouraging health system managers and service providers to make themselves and the system more accountable to communities, improved governance of provincial and district health systems, increased community participation, and improved health service delivery [22]. The model implemented four practices, i.e. cultivating accountability, engaging with stakeholders, setting a shared strategic direction, and stewarding resources responsibly [22]. This involved recruiting more women as community health nursing educators, placing suggestion boxes at health posts, discussing service complaints regular community meetings, and inviting community elders to governance committee meetings [22].

Adams *et al* showed a participatory approach was key to the success of community mental health fora in Sierra Leone [19]. Involving traditional and community leaders helped ensure cultural appropriateness and overcome resistance. For example, community leaders and members were reassured of mental health programme benefits and traditional healers were not threatened as all were involved in goal setting [19].

Erismann *et al’s* examination of community-based health programmes in South Sudan and Haiti discussed engaging service-users in decision-making [25]. Mechanisms to allow communities to propose their own solutions, within established and culturally appropriate structures, need to be implemented to foster capacity-building and increase organisational accountability [25].

Foster *et al* examined community-based distribution of misoprostol for early abortion along the Thailand-Myanmar border, which trained community health workers and a social worker to provide information to Burmese and Karen women about misoprostol and other free medications [26]. Community networks run by health and social workers were successful both because providers were well respected within their communities, and because community knowledge of the project being primarily word-of-mouth from family-members and friends already involved.

Mandal *et al*’s mixed-method study of community health promotion for HIV organised through churches in Sudan, showed this helped reduce stigma but lack of funding ended the project after a year [29]. Elmusharaf *et al*’s examination of pathways to comprehensive emergency obstetric and neonatal care in South Sudan involved community and religious leaders as key informants in design and implementation, to help identify decision-making processes, delays in accessing healthcare, and referral patterns [24].

Ho *et al* used community scorecard methodology in DRC to increase community participation in health facilities’ management, primarily through promoting greater engagement in health facility committees. Community members reported that increased communication with healthcare providers created a more welcoming atmosphere at health facilities, improving the behaviour and attitudes of providers and thus quality of care [27].

#### Co-creation approaches

Only two documents explicitly described the use of elements of community co-design in research or program development, implementation, or assessment, while one described challenges of the absence of any co-creation approach [20,25,33]. Ager *et al*’s examination of health service resilience in Yobe state, Nigeria, in the context of the Boko Haram insurgency, involved community and local government representatives in study design and data interpretation using participatory group model building for systems dynamics analysis [20]. Ager *et al* also described community support as a key factor helping people cope during the insurgency [20]. For example, communities organised transport to help patients access health facilities, while community leaders worked with health-workers to enable clearer understanding of context and potential threats [20].

Sengupta *et al*’s five-year maternal health intervention in tribal areas of India included community-nominated volunteers through group discussions, household surveys, and participatory appraisal to design maternal health services and promotion activities [33]. Intervention staff trained volunteers to implement healthcare protocols, survey methods, advocacy and social mobilisation (e.g. contextual enquiry, persuasive method, participatory rural appraisal, brainstorming), and ethics in their communities [33].

Erismann *et al*’s case study of a community-based health programme in Haiti described how traditional systems of mutual help and support, e.g. *konbit* or rural community work, had been displaced by international aid agencies’ cash-for-work projects, exacerbating the loss of social cohesion through urbanisation [25]. Many Haitians were excluded from decision-making processes and projects were plagued by corruption. This suggested the risk of implementing general ‘aid’ projects without community engagement that are thus based on provider rather than community priorities and needs.

#### Miscellaneous engagement approaches

Nine documents discussed forms of community engagement other than participatory and co-creation approaches [21, 23, 28, 30–32, 34–36].

Akseer *et al’s* study of maternal ‘resilience’ and health system performance in Afghanistan found that building community health-workers’ capacity was key to ensuring continuation of essential healthcare in areas affected by severe conflict, while traditional community *shuras* (councils) were valuable in mediating between non-governmental, government, and armed actors – though intra-group power struggles could derail sustainability [21].

Baingana & Mangan’s study of scaling up of mental health and trauma support among war-affected communities in northern Uganda, found community mobilisation was effective in improving attendance at both mobile and static clinics [23]. Trained village health teams helped mobilise attendance, follow-up patients at home, and check medication adherence and general wellbeing.

Kozuki *et al* found community ownership of an integrated community case management project in South Sudan was weak and Ministry of Health interviewees suggested that community leaders – local chiefs, church leaders, and village elders – should be engaged in its daily administration [28]. Sami *et al*, focused on improving neonatal care in displaced populations in South Sudan, found that using materials in local languages made mothers more receptive to healthcare messages and thus improved interactions [31]. However, some health-workers were frustrated with mothers who rejected modern medicine in favour of traditional beliefs, indicating greater understanding of mother’s perspectives was needed though community health education was recommended [31]. Valadez *et al*, also in South Sudan, found that involving health-workers from local communities in running health promotion initiatives and increasing access to health services, was helpful but necessitated health-workers having security and a strong support system given the risky environment [36].

Rosenberg, studying factors motivating community health-workers to stay in post and their job satisfaction in Liberia, found that the opportunity to serve their community and gain knowledge were major motivators, allowing them to feel proud of their work and want to continue [30].

Saymah *et al’s* assessment of the mental health system in Gaza found that a lack of service-user participation affected healthcare provision [32]. Including service-users in service design and implementation empowered communities and contributed to more effective mental health services

Steven *et al*, studying engagement of rural community leaders (e.g. political, youth, or school) as sexual and reproductive health advocates in DRC, found this was an effective method of addressing communities’ negative opinions of abortion and increasing contraception knowledge [34]. Engaging these leaders, who were at least partly responsible for reinforcing community norms, helped address stigma around these issues and thus improved service-seeking behaviour.

Tangseefa *et al*, examining community engagement in targeted malaria elimination in Karen State, a complex conflict-affected border state in Myanmar, found villagers felt isolated and did not believe health initiatives would benefit them [35]. Community engagement workers disseminated accessible and culturally appropriate intervention information, working with community ‘gatekeepers’ to learn about local norms, describing the intervention, and encouraging participation. This time and effort to respect local norms and leadership increased trust and participation [35].

## Discussion

This review is the first of which we are aware to summarise the primary research literature on community engagement with health systems research and interventions in conflict-affected settings. Despite the recognised importance of community engagement in all aspects of health system functioning [1], we only identified 19 documents that discussed at least one aspect of community engagement, predominantly participatory methods, in diverse conflict-affected countries. Additionally, we found that despite the topic, there was little about what community members themselves described as useful or not, in their own words, within health services. However, the temporal distribution of eligible documents, with most published since 2015, suggests that the significance of community engagement has increased in conflict-affected settings and is becoming a more widely researched phenomenon.

Similarly to Gilmore *et al*, we found that the main community engagement actors, e.g. local leaders, community and faith-based organisations, health committees, and individual stakeholders were engaged primarily in designing and planning, community entry and trust-building, social and behaviour change communication, and logistics/administration [37]. WHO identifies community engagement principles as trust, accessibility, contextualisation, equity, transparency, and autonomy, all of which can contribute to engaging communities in health systems research and interventions [38]. These principles are evident in some of the work we described, including involvement of community leaders (trust and accessibility), contextualisation (e.g. ensuring that conflict, displacement, and other community context is considered), equity (ensuring everyone has opportunities to share their views), transparency (providing a forum for feedback) and autonomy (community bottom-up leadership rather than top-down whenever possible). Three documents included co-design approaches, while nine focused on participatory methods and nine covered more diverse approaches to engagement, such as underlining the importance of community and religious leaders in designing and delivering healthcare [20,24,28,34,35].

Promoting ownership of projects involves building trust, facilitating the construction of shared values, between researchers, practitioners and people in the community, and monitoring the behaviour of all groups involved to ensure that all can participate meaningfully [39]. This is not a simple process, as exemplified by the documents we identified. Co-production of research and practice needs to involve diverse stakeholders and incorporate more perspectives, such as those of policymakers and staff delivering the healthcare. While complex, this results in a richer and more robust project that is more likely to realise its aims [1]. A recent paper suggested that researchers and practitioners should be trained on how to do this effectively at all stages of a project, including an explanation to funders of how this should be done, to promote legitimacy and sustainability of resultant projects [40]. One issue is that community engagement is often talked about but is framed as a consultative process rather than an attempt to empower communities, with the aim of putting final decision-making in their hands [41].

While involving community leaders, who are perceived as having high status, is important in encouraging community engagement in initiatives, it is also vital to ensure that community groups are also represented in health system research and practice. Marginalised groups may not be able to share their views with or through community elites but must be included in an equitable health system [42]. One recent rapid review of community-engaged responses to the Covid-19 pandemic found that inclusion of marginalised groups in participatory action was facilitated by using local languages to create a safe space where they felt able to express themselves, removing the stigma of being seen as ‘victims’ and instead being taken seriously as ‘actors’ with their own concerns [43]. Nevertheless, many documents we identified underlined the importance of working with communities to produce culturally acceptable interventions that did not threaten existing community power relations, as demonstrated in promoting mental health in Sierra Leone [19]. Involving community leaders in a programme addressing what is a highly sensitive issue in many cultures ensured that members of the community did not feel stigmatised about participating. Involving groups to mediate between governments, non-governmental organisations and armed groups, as was shown in Afghanistan, helped health-workers continue working in conflict-affected areas [21].

Allowing communities to provide feedback on healthcare provision, and a safe space to discuss their concerns without fear of retribution or harm, was important in tailoring healthcare provision in countries as diverse as Afghanistan [22] and DRC [27]. Similarly, in Darfur, a women’s group involving displaced and host-community women promoted discussions on common health issues and potential solutions in a safe space [44]. It is essential that communities are provided with evidence that programmes and initiatives are working. This involves ensuring that communication channels are both appropriate and effective. However, this can be difficult – for example, in Afghanistan, the volatile situation, with opposition groups in conflict with the government, hospital staff abducted, and a lack of security, made it difficult to engage with staff and patients in healthcare facilities [22]. Similarly, in DRC, a lack of accountability, transparency and any mechanism to sanction healthcare providers for poor performance meant that providing feedback was seen as ineffective, even where it was possible to do so [27]. Health promotion methods need to have built in flexibility to be able to adapt to the nature of conflict-affected settings. Community leaders may change frequently and consistent funding and sustainability are unlikely. To build strong partnerships with communities, we must take into account health system determinants such as availability (or otherwise) of resources, strong governance from community leaders, social, religious and cultural factors, and political will. Future research should identify ways of engaging healthcare service-users in conflict-affected settings to provide optimal healthcare provision for these communities, and ensure that their perspectives and contributions are incorporated into any research findings and initiatives.

### Limitations

Several limitations should be considered. Only sources with an English abstract were included, which means some relevant documents in other languages may have been excluded. Similarly, we may have missed documents that were only indexed in databases we did not search. We did not appraise the quality of our sources, as this scoping review was intended to identify as broad and diverse a range of eligible documents as possible, unlike a more narrow systematic review approach.

### Conclusion

Engaging communities in identifying and setting priorities, decision-making, and implementing health system interventions and research can give the public an opportunity to share their views and a sense of ownership in the services they use, and should thus make healthcare provision more successful. Community engagement is particularly important in conflict-affected and high-risk, insecure settings, where priorities may be difficult to identify, and other factors, such as security, power relations, and structural inequalities must be considered. However, despite its importance we found limited research literature on community engagement in conflict-affected settings.

## Author contributions

NH conceived the study, with inputs from ADB and MM. All co-authors contributed to title/abstract and full-text screening. ADB and MM extracted and synthesised data with inputs from all authors. ADB drafted the manuscript. NH revised for critical content. All authors contributed to interpretation of findings and approved the version for submission.

## Paper context

Healthcare research, planning, and delivery with minimal community involvement results in financial wastage, failure to meet objectives, and frustration in the communities that programmes are designed to help. Involving communities in setting priorities, decision-making, and implementing solutions encourages ownership, which increases healthcare interventions’ success. Engaging communities can be particularly difficult in conflict-affected settings, where priorities may be difficult to identify, and many other factors, such as safety, power relations, and entrenched inequalities, must be considered.
